# Suboptimal decision making and interpersonal problems in ADHD: longitudinal evidence from a laboratory task

**DOI:** 10.1038/s41598-024-57041-x

**Published:** 2024-03-19

**Authors:** L. Sørensen, S. Adolfsdottir, E. Kvadsheim, H. Eichele, K. J. Plessen, E. Sonuga-Barke

**Affiliations:** 1 Department of Biological and Medical Psychology, University of Bergen, Jonas Liesvei 91, 5009 Bergen, Norway; 2 Division of Vision Impairments, Statped – National Service for Special Needs Education, Bergen, Norway; 3 Department of Clinical Medicine, University of Bergen, Bergen, Norway; 4 Regional Resource Centre for Autism, ADHD and Tourette Syndrome Western Norway, Division of Psychiatry, Haukeland University Hospital, Bergen, Norway; 5 Division of Child and Adolescent Psychiatry, Department of Psychiatry, Lausanne University Hospital, University of Lausanne, Lausanne, Switzerland; 6 Department of Child and Adolescent Psychiatry, King’s College London, London, UK; 7 Department of Child and Adolescent Psychiatry, Aarhus University, Aarhus, Denmark; 8 Department of Psychology, Hong Kong University, Hong Kong, China

**Keywords:** Risk factors, Signs and symptoms, Neuroscience, Psychology

## Abstract

Over half of children with Attention-Deficit/Hyperactivity Disorder (ADHD) display interpersonal and social problems. Several lines of research suggest that suboptimal decision making, the ability to adjust choices to different risk-varying options, influences poorer choices made in social interactions. We thus measured decision making and its prediction of social problems longitudinally with the Cambridge Gambling Task in children with ADHD over four years. Children with ADHD had shown suboptimal decision making driven mainly by delay aversion at baseline and we expected this to be a stabile trait which would predict greater parent-reported social problems. From the baseline assessment (n = 70), 67% participated at the follow-up assessment, 21 from the ADHD group and 26 from the typically developing group. The mean age at the follow-up was 14.5 years old. The results confirmed our expectations that suboptimal decision making was a stabile trait in children and adolescents with ADHD. Although delay aversion did not differ from controls at follow-up it still proved to be the main longitudinal predictor for greater social problems. Our findings indicate that impulsivity in social interactions may be due to a motivational deficit in youth with ADHD.

## Introduction

Over half of the individuals with attention-deficit/hyperactivity disorder (ADHD) have difficulties in developing and maintaining social relationships^1–4^. These interpersonal problems seem to be the result of ill-considered and poorly timed social interventions and responses rather than a lack of knowledge about appropriate social conduct^1,5,6^. For instance, people with ADHD might choose to interject a comment into a conversation that has an inappropriate content, at an inappropriate moment or in an inappropriate way without thinking through the consequences of the actions^7,8^. In this sense interpersonal problems can be conceptualized, from one perspective, as expressions of suboptimal decision making causing poorer and impulsive choices to be made in social situations^9^. However, the question of whether the social and interpersonal problems of people with ADHD are underpinned by more general deficits in basic decision-making skills has not yet been addressed. Investigating the role of decision making in social problems in ADHD could lead to a complementary understanding compared to simply focusing on that inattentive, impulsive, and hyperactive people cannot focus, wait, or sit still enough to develop successful interactions. The core ADHD symptoms are in general found to predict social problems^10^, at the same time as, treatments that are effective in reducing ADHD symptoms, such as psychostimulant medication, only have small effects on enhancing social skills^11–14^. This suggests a dissociation between the core symptoms of ADHD and other neurocognitive processes linking the diagnosis to social impairment. It is thus a call for exploring if processes, such as social decision making, might improve the conceptualizations of the causes for social problems in ADHD and as such, contribute to finding relevant targets for social skill training^15^.

In the current paper, we examined this question by exploring the prospective predictive relationship between children’s performance on a widely used and well-validated task measuring risky and impulsive decision making (Cambridge Gambling Task; CGT)^16,17^ and parent-reported social and interpersonal problems later in development. This task measures the extent to which individuals can adjust their choices to more-or-less-risky options by integrating external information with internal value systems. The choices are made between response options with different outcome probabilities^16,18^. Suboptimal decision-making was measured with the CGT scores of: (1) Risk adjustment (the difficulty in adjusting decisions according to level of risk by learning from previous choices), (2) delay aversion (the choice of the less delayed option rather than the choice with the highest return), (3) reflection time (shorter reflection times reflect poorer inhibitory control^19^), and (4) risk proneness (over-attraction to the risky options). In phase one of the study, we included a cross-sectional baseline analysis of 36 drug-naïve children with ADHD and 34 typically developing peers aged between 8 and 12 years old. We observed that ADHD was associated with fewer optimal decisions (poorer risk adjustment), which was driven primarily by higher levels of delay aversion^20^. This finding was consistent with much broader literature highlighting delay aversion as a core motivational component of ADHD across settings^21–25^ and further, that poorer risk adjustment in ADHD seems to be due to other processes than being risk prone per se^26^. These different decision making parameters may predict greater social problems as part of an overall suboptimal decision making. For instance, impulsive choices made in social settings have been suggested to be caused predominantly by poorer inhibitory control^27,28^ or delay aversion^29,30^. However, several studies have shown a weak link between inhibitory control and social problems in ADHD^31–35^. As far as we know, no study has investigated the link between delay aversion and social problems despite the suggestions that it leads to impulsive and disruptive behavior in subjectively experienced stimulus-poor environments^29,30^. Furthermore, ADHD has been associated with increased risk prone behavior (see^36^). It is not clear to what extent this risky behavior in youth with ADHD is linked to a different social functioning in comparison with typically peers. One recent study indicated that peer influence compared to no such influence increased risk prone choices during the Balloon Analogue Risk Task across the groups of adolescents with ADHD and their typically developing peers alike^37^. This may suggest that risk proneness is similarly related to social influence in ADHD as in non-ADHD.

We followed up on the sample after 4 years to study the developmental outcomes associated with suboptimal decision making in ADHD. Based on the hypothesis that interpersonal problems in ADHD are driven by core deficits in decision making, we predicted that at follow-up individuals with ADHD displaying suboptimal decision making at baseline (T1) would (i) show more social and interpersonal difficulties and (ii) continue to display poor risk adjustment and delay aversion on the CGT and at the same time continue to not show difference from typically developing peers in reflection time (inhibitory control) or risk proneness. In addition to parent-reports of social problems from the Child Behavior Checklist (CBCL; Achenbach and Rescorla^38^), we also included parent-reports on two other subscales from CBCL of conduct problems and anxiety/depression problem symptoms due to these problems often being comorbid with ADHD and recognized in accompanying problems in social interactions. Children with ADHD often have social problems due to high frustration levels affecting their social functioning^39^. Conduct problems characteristically reflect higher levels of irritability, delinquency, and aggressiveness in the behavior towards peers and others^40^. Furthermore, problematic symptoms of anxiety and depression are associated with higher frustration levels and irritability^41^ as well as with social isolation^42^.

## Results

### Sample characteristics

From the original sample (N = 70) at baseline performing the CGT (T1), 67% (N = 47) participated at the follow-up assessment (T2); 58% (n = 21) from the ADHD group and 77% (n = 26) from the typically developing group (see Supplemental Fig. 1). At T2, the age range was 11–17 years old with a mean age of 14.5 (SD = 1.31). The two groups did not differ significantly in age or gender distribution (see Table 1). The mean T1 and T2 interval was 4.5 years (SD = 0.7) with no significant difference in interval between the two groups. At T2, all the adolescents in the ADHD group still met the criteria for an ADHD diagnosis, and 48% of these had a comorbid disorder; six had anxiety; six had oppositional defiant disorder (ODD), two had tics, one had depression, and one obsessive–compulsive disorder. In the control group, two had an anxiety disorder. In the ADHD group, 76% (n = 16) used CNS stimulants to treat their ADHD. Of these, 88% conducted a washout period of at least 24 h before testing, whereas two participants had a washout period of at least 12 and 18 h, respectively. These are all acceptable washout periods due to the two that had a shorter period used methylphenidate, which has a half-life of two to three hours and the active ingredient eliminated after 10–15 h^43,44^. Children with ADHD who remained in the study at T2 showed no significant differences in scores on the CGT, Full-Scale IQ, or ADHD symptoms at T1 compared to their counterparts who dropped out (see Supplemental Table 1).

**Table 1:** Demographic information, CGT parameter scores, and social problem scores in the groups of adolescents with ADHD and healthy controls.

Variables	ADHD (n = 21)				Controls (n = 26)				Group analysis	
	T1		T2		T1		T2		T1	T2
	Mean	SD	Mean	SD	Mean	SD	Mean	SD	*t*	*t*
Age	9.88	1.25	14.33	1.50	10.08	1.00	14.58	1.15	0.20	0.64
T1–T2 interval			4.46	0.91			4.5	0.56		0.21
Full-scale IQ	94	7.03			109	10.42			5.70**	
Risk adjustment	− 0.54	0.73	− 0.10	0.77	− 0.00	0.92	0.50	1.13	2.19*	2.06*
Delay aversion	0.80	0.99	− 0.36	0.81	0.04	0.91	− 0.39	0.88	− 2.74**	− 0.10
Reflection time^^^	0.61	1.14	− 0.47	0.59	0.58	0.68	− 0.75	0.39	− 0.08	− 1.97
Risk proneness	− 0.00	0.99	0.29	0.82	0.26	1.17	− 0.26	0.95	0.09	− 2.11*
Social problems	80.10	16.24	81.48	13.34	53.92	7.97	53.88	9.72	− 7.23**	− 8.20**
Anx./dep. sympt	69.00	18.90	77.38	18.89	57.58	14.34	58.35	13.91	− 2.36*	− 3.98**
Conduct problems	65.43	8.91	63.33	11.33	50.85	1.74	51.85	5.27	− 8.18**	− 4.60**
Sex	*n* males		*n* females		*n* males		*n* females		*X2*	
	15		6		15		11		9.49	

*DM* decision making, *anx.* anxiety, *dep.* Depression, *sympt*. symptoms, ^^^inhibitory control. The CGT parameters are in z scores and the social problem scores are in percentile scores. **p < 0.01; *p < 0.05.

### Did decision making deficits seen at baseline persist to follow up?

**Figure 1:**
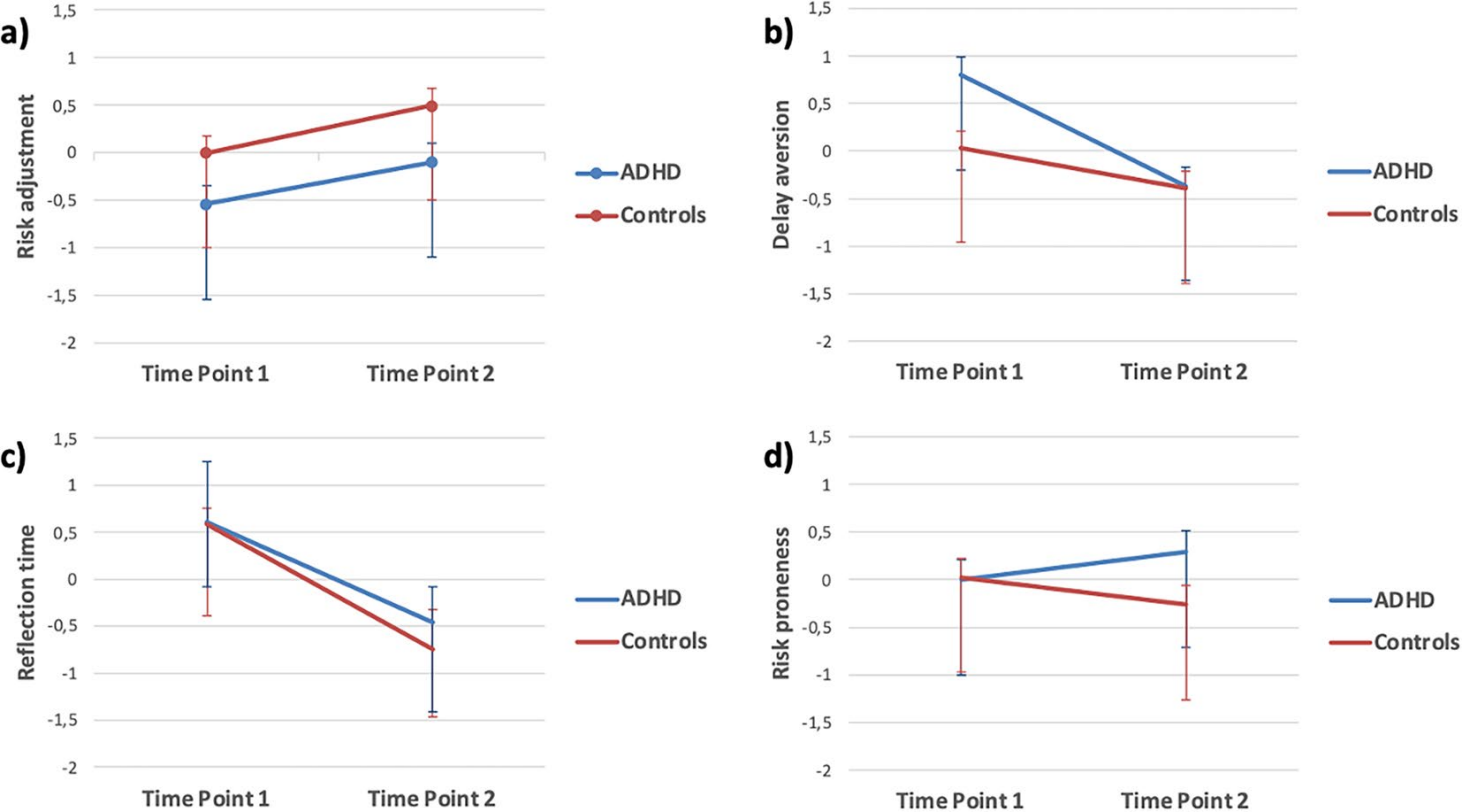
Group effects shown at baseline and follow-up on the CGT parameters of (**a**) risk adjustment, (**b**) delay aversion, (**c**) reflection time (inhibitory control), and (**d**) risk proneness.

Cross-sectionally, the ADHD group showed poorer risk adjustment compared with the control group at both T1 and T2, higher delay aversion only at T1, and higher risk proneness only at T2 (see Table 1). No cross-sectional group differences appeared in relation to reflection times (inhibitory control). Longitudinally (alpha levels (α) were Bonferroni corrected for conducting four ANOVAs; p < 0.013), including both ADHD and the two time points as factors, there was a main effect (*F*(1,90) = 8.91, *p* = 0.004, *η*_*p*_^2^ = 0.09) of group on decision making quality with the ADHD sample showing poorer risk adjustment at both baseline and follow-up (see Fig. 1 and Supplemental Table 2). There was no significant effect of time and no interaction between time and group. Furthermore, the risk adjustment scores at baseline and follow-up were significantly correlated in the whole sample (*r* = 0.36, *p* = 0.014) and in the ADHD group (*r* = 0.51, *p* = 0.018) but not in the control group alone (*r* = 0.20). However, stability was less clear for the other decision elements with the ADHD group showing more delay aversion than controls at baseline than follow-up (main effect of time points: *F*(1,90) = 18.16, *p* = 0.001, *η*_*p*_^2^ = 0.20). Still, in the whole sample (*r* = 0.34, *p* = 0.02) and in the ADHD group (*r* = 0.45, *p* = 0.041), greater delay aversion at both time points correlated significantly, except in the control group (*r* = 0.29). No significant effect of ADHD appeared on risk proneness or in reflection times (inhibitory control) neither at baseline nor follow-up when the two time points were included as a factor in addition to ADHD. Risk proneness as measured at the two time points did not correlate, whereas reflection times correlated highly between the two timepoints in the whole sample and in both subgroups. See Supplemental Table 4 for all the intercorrelations of the CGT parameters cross-sectionally and longitudinally.

### Did adolescents with ADHD show more social problems?

Parents reported that children with ADHD showed greater social problems and greater problems of conduct behavior and anxiety/depression at both time points compared with control children (see Table 1). At follow up all three problem areas of social problems, conduct behavior, and anxiety/depression correlated highly with each other (see Table 2).

**Table 2:** Bivariate correlations between the CGT parameters at both time points and parent-reported social problems and conduct and anxiety/depression problems at follow up.

	T2 social	T2 anxiety/	T2 conduct
	problems	depression	problems
T2 anxiety/depression	0.60**		
T2 conduct problems	0.67**	0.56**	
T1 CGT risk adjustment	− 0.32*	− 0.23	− 0.14
T1 CGT delay aversion	0.43**	0.40	0.22
T1 CGT reflection time^^^	− 0.11	− 0.03	0.31*
T1 CGT risk proneness	− 0.20	− 0.28	0.01
T2 CGT risk adjustment	− 0.40**	− 0.37*	− 0.22
T2 CGT delay aversion	0.02	0.17	− 0.29
T2 CGT reflection time^^^	0.33*	0.27	0.20
T2 CGT risk proneness	0.16	0.006	− 0.05

**p < 0.01; *p < 0.05. *DM* decision making, ^^^Inhibitory control.

### Were social problems correlated with decision making on the CGT cross sectionally and longitudinally?

**Figure 2:**
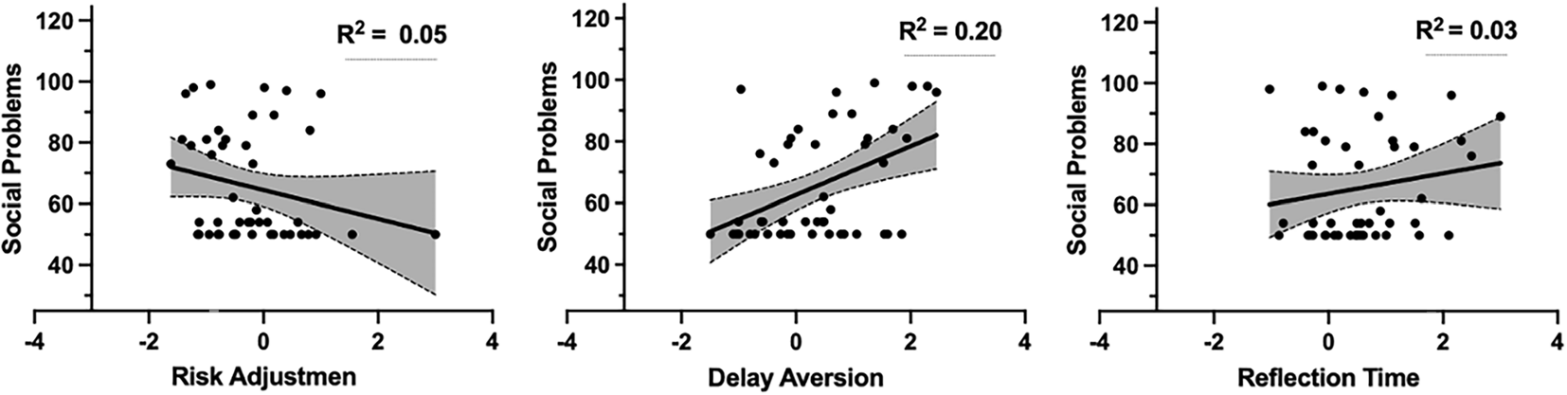
Scatterplots of the longitudinal relationship between the baseline CGT parameters of risk adjustment, delay aversion, and reflection time (inhibitory control) and parent-reported social problems at follow-up. Delay aversion was the only predictor of these CGT parameters that predicted significantly greater social problems at follow-up.

Table 2 shows the correlations between social problems at follow-up and CGT parameters at baseline and follow-up. Cross-sectional associations at follow-up were significant between the CGT parameters of poorer risk adjustment and longer reflection times (inhibitory control), and further, between poor risk adjustment and greater social problems, and greater anxiety/depression problems. Furthermore, the CGT parameters did not correlate with conduct problems nor did risk proneness correlate with social problems or problems of conduct behavior and anxiety/depression at follow-up. Longitudinally, significant correlations were seen for both the baseline CGT parameters of poorer risk adjustment and greater delay aversion with greater social problems at follow-up. Baseline reflection times (inhibitory control) and risk proneness did not correlate with social problems at follow-up. Further, only the baseline CGT parameter of greater delay aversion correlated with higher anxiety/depression problems at follow-up, and only longer reflection times (inhibitory control) at baseline correlated with greater conduct problems at follow up. To test for the specificity of the effect baseline CGT parameters had on social problems and problems of conduct behavior and anxiety/depression at follow up, we included the three baseline CGT parameters that correlated longitudinally with these problems as predictors (risk adjustment, delay aversion, and reflection times) in three multiple linear regression analyses with the three CBCL scores as outcome variables [alpha level (α) was Bonferroni corrected for conducting three regression analyses; p < 0.017]. The results showed that only greater delay aversion at baseline, and not poorer risk adjustment or reflection times (inhibitory control), predicted greater social problems at follow up (see Table 3 and Fig. 2). None of the CGT parameters specifically predicted level of conduct problems or anxiety/depression problems at follow-up.

**Table 3:** The longitudinal prediction of baseline CGT parameters of poorer risk adjustment, delay aversion, and reflection time (inhibitory control) on parent-reported social problems and conduct and anxiety/depression problems at follow-up.

Predictors	Adj. R^2^	df	F	p	B	β	t	p	95% CI for B
T2 parent-reported social/interpersonal problems from CBCL									
T1 risk adjustment	0.17	3/43	4.14	0.012*	− 2.52	− 0.12	− 0.80	0.430	− 8.89 to 3.86
T1 delay aversion					6.99	0.40	2.59	0.013*	1.54 to 12.43
T1 reflection time^^^					2.78	0.14	1.00	0.325	− 2.85 to 8.40
T2 parent-reported anxiety/depression problems from CBCL									
T1 risk adjustment	0.11	3/43	2.84	0.049	− 1.56	− 0.07	− 0.45	0.653	− 8.47 to 5.36
T1 delay aversion					6.87	0.37	2.34	0.024	0.96 to 12.79
T1 reflection time^^^					0.17	0.01	0.06	0.957	− 5.93 to 6.27
T2 parent-reported conduct problems from CBCL									
T1 risk adjustment	0.10	3/43	2.73	0.055	0.44	0.04	0.23	0.817	− 3.34 to 4.22
T1 delay aversion					2.78	0.28	1.73	0.09	− 0.45 to 6.01
T1 reflection time^^^					3.93	0.35	2.38	0.022	0.60 to 7.26

*Bonferroni corrected p-level (0.05/3) = 0.017. *DM* decision making; ^^^Inhibitory control.

## Discussion

**Figure 3:**
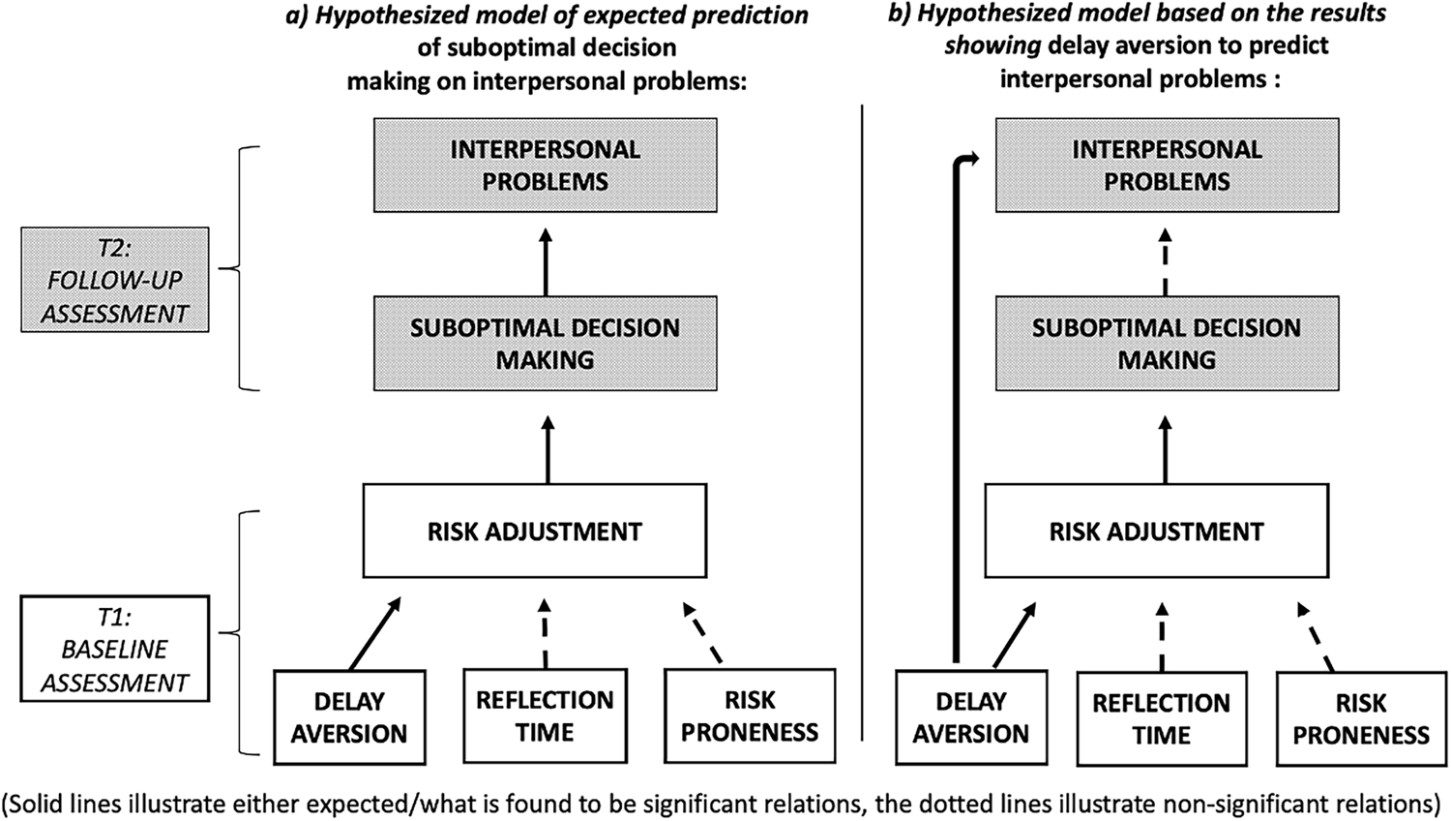
Hypothesized models illustrating (**a**) the expected prediction of baseline suboptimal decision making on longitudinal interpersonal problems, and the effect of delay aversion on interpersonal problems via poorer risk adjustment, and (**b**) the role of baseline delay aversion both as contributing to suboptimal decision making and directly predicting interpersonal problems four years later.

In the current study, children with ADHD were followed up longitudinally to study the development of suboptimal decision making over time and its prediction of social problems between childhood and adolescence. We originally found that the children with ADHD displayed suboptimal choices as measured with risk adjustment at baseline, driven primarily by delay aversion on the CGT compared with typically developing peers. At follow-up four years later, we expected that individuals with ADHD would still show poorer risk adjustment and greater delay aversion than their typically developing peers, and that baseline suboptimal decision making would predict greater parent-reports of social problems and conduct and anxiety/depression problems. As expected, the results showed that poorer risk adjustment was a stable trait over 4 years in children and adolescents with ADHD. Delay aversion, on the other hand, was no longer significantly different at follow-up in the ADHD group compared with the control group. Still, greater delay aversion along with poorer risk adjustment correlated from baseline to follow-up only in the ADHD group and not in the control group. The CGT parameters of poorer risk adjustment, greater delay aversion, and longer reflection times (inhibitory control) were correlated both cross-sectionally and longitudinally with greater interpersonal problems and conduct and anxiety/depression problems. However, when including these CGT parameters in the same statistical model, greater delay aversion at baseline (T1) was the only CGT parameter that four years later (T2) predicted greater social problems (see Fig. 3). Furthermore, none of these CGT parameters at baseline predicted conduct or anxiety/depression problems four years later. Risk proneness was not different in the ADHD group at T1 but showed a tendency to be higher in this group at T2. It did, however, not correlate with social problems or conduct or anxiety/depression problems. Reflection time (inhibitory control) was not different between the groups at either time point.

Parents and teachers consistently report that children with ADHD struggle with interpersonal interactions and relationships^1,4,6,35^, as is the case in the current study. These reports typically coincide with the perception reported by peers that children with ADHD are more often rejected and neglected by peers, having fewer friends and fewer and less diverse social activities^13,31,45,46^. Our results indicate that these problems are predicted by difficulties in adapting dynamically to changing contextual conditions. On the CGT this can be observed by difficulties in adapting choices to the changing patterns of outcome probabilities (poorer risk adjustment). In the current study, the motivational style of escaping delays was the main driver for these problems. Delay aversion has previously been suggested to cause social problems by leading to impulsive and disruptive behavior^29,30^. Importantly, our study appears, as far as we know, to be the first to experimentally investigate and link delay aversion with greater interpersonal problems. This is in line with the dominant causal hypothesis of ADHD in children in that they tend to choose immediate rewards more consistently when this choice gives escape from delay compared to when the same choice does not escape delay^23^. This drive for escape when children with ADHD perceive situations as tedious (stimulus-poor) is suggested to be associated with a negative affective state (high frustration), which is supported by fMRI studies of adolescents^25,47^ and adults^48^ with ADHD.

In line with findings from several previous studies^31–35^, the children with ADHD did not show suboptimal decision making or social problems due to poorer inhibitory control as reflected in shorter reflection times. Rather, it was a general finding that longer reflection times correlated with greater social problems, which probably are linked to challenges of processing information fast enough in social situations^49^. Important to note, though, is that a previous study has shown that the delay aversion score from the CGT can be challenging to distinguish from poor inhibitory control in ADHD^22^. In our original study^20^, we therefore tested for this and found that (a) the delay aversion score was linked to shorter test duration time—showing the CGT delay aversion score to be linked to escape of delay and not just an impulsive drive for immediate reward (see^23,50^), and (b) the prediction of delay aversion in explaining suboptimal decision making in ADHD was not explained by poorer inhibitory control (as measured with the Cambridge Stop Signal Test) or by level of intelligence and working memory capacity. Inhibitory control, intelligence and working memory capacity did not either covary in our original study with suboptimal decision making when testing the group differences between ADHD and control children^20^.

Our findings showed that delay aversion at baseline tended to specifically predict greater interpersonal problems in general and not conduct or anxiety/depression problems. Both conduct and anxiety/depression problem scores were significantly higher in the ADHD group and highly correlated with social problems. In relation to the CGT scores, greater delay aversion correlated with higher anxiety/depression problem symptoms, and longer reflection times with greater conduct problems. This supports delay aversion being described as expressing negative affectivity^48^—typically associated with higher levels of anxiety and/or depression^51^. Longer reflection times have also been found to be associated with lower motivation to do tasks as instructed^52^, which higher conduct problems can reflect via non-compliance with external expectations. However, including the CGT parameters that correlated with social problems and the problem scores of conduct behavior and anxiety/depression in the same regression models, showed that none of them specifically predicted higher conduct or anxiety/depression problems.

To our knowledge, this is the first study that has investigated the link between suboptimal decision making and interpersonal problems in ADHD. However, in adults with schizophrenia^53^ and in young healthy adults^54^, both studies using a different test method than CGT, found that suboptimal decision making was associated with poorer social skills and interpersonal relationships. In children with ADHD, one study investigated the ability of teacher-reported ADHD symptoms to predict parent-reports on the social problem subscale from the CBCL and performance on a decision making task using affective cues two years later^55^. The ADHD symptoms predicted both greater social problems and poorer decision making. However, the cross-sectional correlation coefficient between social problems and the affective cuing decision making task scores was low (r = 0.21). Future studies are encouraged to further test the prediction of suboptimal decision making on social problems in ADHD. The low sample size in the current study may rise issues of low statistical power when testing longitudinal associations despite the a priori power analysis showed the sample size to be sufficient for avoiding a type II error.

In the current study, the children with ADHD were drug naïve at baseline and the majority on medication at follow-up. This may have affected the longitudinal findings in that medication according to its objectives is supposed to have long-term effects on cognition and motivational style^56^. These effects may be present even though we asked the participants to refrain from taking the medicine before the testing. We found, however, that suboptimal decision making was a stable trait in the ADHD group suggesting that CNS medication did not have a long-term effect on improving the ability to choose optimal options in ambiguous and risky situations (on the gambling task)—besides the short-term effects shown previously on the CGT^57^. This is in line with a recent study on the effects of methylphenidate in adolescents with ADHD, in which no long-term effects were found on any of the outcome measures including on cognitive functioning^14^. It is possible that medication may have led to a higher tolerance for delay at the follow-up assessment, or it may be due to the children with ADHD being older and more mature in their decision making abilities. In relation to social competence and the effect of CNS medication, one systematic review found that children treated with medication and/or non-pharmacological interventions for their ADHD had better social functioning compared to untreated children with ADHD^58^. Other studies, however, have found that methylphenidate alone, or in combination with cognitive behavioral therapy, had only a limited effect on social behavior in children with ADHD^11–14^. In boys with ADHD, the positive effect of methylphenidate was specifically observed in higher compliance with expected behavior and a decrease in aggressive behavior tendencies^12^.

## Conclusion

Interpersonal problems are recognized as one of the important impairments negatively affecting quality of life in children with ADHD^58,59^. Early predictors and mechanisms of interpersonal problems are thus essential to identify^15^. The current study showed that suboptimal decision making driven by delay aversion can be an important predictor to address in future studies of interpersonal competence in ADHD. A deeper understanding of this relationship may initiate specific programs of psychoeducation about living with ADHD for caregivers and the children. This relationship may also be important to address in training programs to improve quality of life for children with ADHD.

## Methods

### Participants

The current study was a follow-up of children 8 to 12 years old with ADHD and age-matched typically developing peers performing the CGT^20^. The children were originally included at baseline (T1) with a Full-Scale Intelligent Quotient (FSIQ) > 80^60^ and diagnostically evaluated with the Schedule for Affective Disorders and Schizophrenia for School-Age Children—Present and Lifetime Version (K-SADS)^61^. The K-SADS was re-administered at the follow-up assessment (T2). The test administrators of the neuropsychological test battery including the CGT were blinded to group status both at T1 and T2. The parents reported on the children’s’ mental health and social functioning at both time points (described below). At T2, children were first interviewed with the K-SADS while parents filled out questionnaires, and subsequently the children performed a neuropsychological test battery and filled out questionnaires while the parents were interviewed with the K-SADS. The study protocol was approved by the Regional committee for medical research ethics of western Norway (study number: 2014/1304) and research was conducted according to relevant regulations and guidelines. Informed consent was given both orally and in written form from all the parents and the adolescents. Both the adolescents’ (at T2) and their parents (at T1 and T2) signed written consent in accordance with the Declaration of Helsinki. The participants received a reimbursement of $115.

### Cambridge gambling task (CGT)

**Figure 4:**
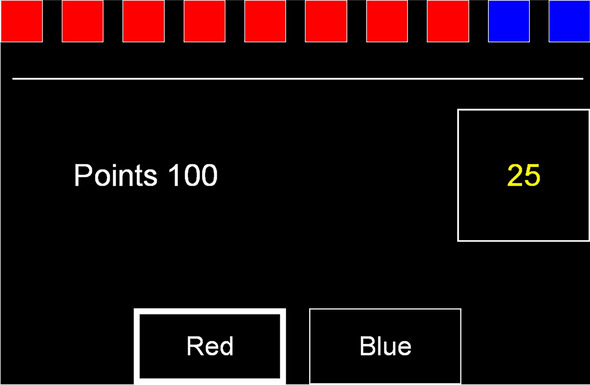
A screen shot of the Cambridge Gambling Task (CGT). The red and blue boxes at the top of the screen are hiding a yellow token and the red and blue squares at the bottom of the screen are pushed to guess which colored box the token is hidden behind. The numbers presented represent percentages of the points displayed at the left-hand side that are presented either in ascending or descending sequences. Note. The image is printed with permission from © Copyright 2023 Cambridge Cognition Limited. All rights reserved.

The CGT from the Cambridge Neuropsychological Test Automated Battery, CANTAB; www.camcog.com^16,17^, was administered at T1 and T2 (see Fig. 4). The children were first instructed that a yellow token was hidden behind either a blue or red box, presented in an array of 10 boxes at the top of the computer screen. Secondly, they were instructed to make a bet on the likelihood of their decision being correct or not. Points were presented in a box on the right-hand side in 5-s increments/decrements sequences. The children touched the box to place a bet. In four of the test blocks, the bets were presented in an ascending order of 5, 25, 50, 75, and 95% portion of the points that the children would “earn” on each trial (displayed on the left-hand side of the computer screen), and in the other four blocks they were presented in a descending order (95, 75, 50, 25, and 5% portion of the points). The order of presentation (ascending or descending) was counterbalanced across individuals within the groups at both time points. The task was administered on a desktop PC and responses recorded via a touch-sensitive screen (see Sørensen et al.^20^ for further details). As measures of suboptimal decision making, we included the CGT generated scores of (a) risk adjustment measuring the ability in adjusting decisions according to level of risk by learning from previous choices, (b) delay aversion measured the proportion of choices made for the option available most immediately irrespective of the outcome, (c) reflection time (inhibitory control) measured the time taken to think about the options during the decision phase and was calculated based on the deliberation time between stimulus presentation and choice outcome, and (d) risk proneness was defined by the total number of points that were gambled on the most improbable outcome—reflecting the overall tendency to take risks. These scores were centralized as z-scores.

### Social problems and conduct and anxiety/depression problems

Parents completed the Child Behavior Checklist 6–18 (CBCL)^38^ at T1 and T2. The CBCL is a 113-item rating scale with a 3-point response scale, from 0 for not true to 2 for very true/often true. The CBCL is a widely used instrument with excellent test–retest reliability, internal consistency, and interrater reliability^38^. The total score from the subscale of social problems was used as a measure of interpersonal problems. The items of this subscale relate to different areas of social functioning including peer rejection, interaction style, impact of peer rejection (feeling lonely), and behaviors that are observed with peer rejection such as clumsiness and speech problems. The subscales of social problems, anxiety/depression problems, and conduct problems have all been shown to have high test–retest reliability^38^ and acceptable internal consistency (≥ 0.70) with the Norwegian translation^62^. The standardized percentile scores were included in the statistical analyses.

### Statistical analyses

All analyses were performed with SPSS, version 28. The longitudinal sample was first described by testing group differences on sample characteristics with independent sample t-tests. To test for longitudinal effects of ADHD on decision making, four analyses of variances (ANOVAs) were conducted with a 2 (ADHD vs. no ADHD) × 2 (effects of timepoint vs. no effects of timepoints) factorial design to test for between-group differences on the CGT scores of risk adjustment, delay aversion, reflection time (inhibitory control), and risk proneness. An interaction effect between ADHD and time points would suggest that the effect of ADHD on one of the CGT scores would be specific to one of the time points. Age and gender were not included as covariates in the final ANOVA models due to not changing the significant effects of ADHD on the CGT scores. See Supplemental Table 3 for the age-adjusted ANOVA results in which age only covaried with delay aversion and gender only with risk proneness (see also Supplemental Tables 4 and 5). Further, the effect of ADHD on social problems and problems of conduct behavior and anxiety/depression was investigated with independent sample t-test analyses. Bivariate correlations were estimated first on the relationship between the parent-reported problem scores and thereafter on the cross-sectional and longitudinal relationship between the CGT scores and the parent-reported scores of social problems, conduct problems, and anxiety/depression problems. The baseline CGT scores (T1) that correlated with the social and conduct and anxiety/depression problems at follow-up (T2) were further included (simultaneous) as predictors in three multiple linear regression analyses with the follow-up parent reported scores on social problems, conduct problems, and anxiety/depression problems as the outcome variables, respectively. Age was not included as a covariate in the final linear regression models due to not covarying with the parent-reported outcome scores of social problems, conduct problems, or anxiety/depression problems (see Supplemental Table 6 for the bivariate correlations between age and the CBCL scores of social problems, anxiety/depression problems, and conduct problems).

We adjusted for multiple analyses by using Bonferroni correction of alpha level (α) in the between-group analyses (ANOVAs); p = 0.05/4, (four CGT outcome scores) which gives an α corrected p level of 0.013, and in the multiple linear regression analysis with parent-reported problem scores as the outcome variables; p = 0.05/3 (three CBCL outcome scores), which gives an α corrected p level of 0.017. Outliers were defined using a ± 3 standard deviations threshold from the sample mean and replaced and included in the statistical analyses with a score of ± 3 standard deviations from the sample mean. On the CGT: One child with ADHD showed very long reflection time (z = 4.24) and two typically developing children showed very high scores on risk adjustment (z = 3.18 and z = 3.52). No outlier scores were detected on the CBCL.

A priori estimation of statistical power was performed to determine the sample size needed to estimate longitudinal statistical effects of ADHD on suboptimal decision making. Using g*power, we included the mean scores of risk adjustment of the ADHD group (0.61) and the controls (1.20), respectively, from T1^20^, and adjusted for the statistical variance (SD = 0.65) of the ADHD group. We specified the conventional level of sufficient power to be 1 − ß = 0.80 and a significance of α = 0.05. The analysis revealed that a total number of n = 42 young people would at least be required to avoid a type II error in our study, which is below our sample size of n = 47.

### Supplementary Information

Supplementary Information.

## Data Availability

The datasets used and analyzed during the current study are available from the corresponding author on reasonable request.
