# Supplementary material for: Suboptimal decision making and interpersonal problems in ADHD: longitudinal evidence from a laboratory task
Source: Sci Rep. 2024 Mar 19;14:6535. doi: 10.1038/s41598-024-57041-x (PMC10951300; doi:10.1038/s41598-024-57041-x)
Supplement: Supplementary file 1 — Supplementary Information. [file 41598_2024_57041_MOESM1_ESM.pdf]

Supplemental Information:

Suboptimal decision making and interpersonal problems in ADHD: Longitudinal evidence from a laboratory task.

Sørensen[1*], L., Adolfsdottir[2], S., Kvadsheim[3], E., Eichele[4], H., Plessen[5], K.J., & Sonuga-Barke[6], E.

[1]Department of Biological and Medical Psychology, University of Bergen, Bergen, Norway
[2]Division of Vision Impairments, Statped – National Service for Special Needs Education, Bergen, Norway
[3]Akershus University hospital, Lørenskog, Norway
[4]Regional resource centre for autism, ADHD and Tourette syndrome Western Norway, Division of Psychiatry, Haukeland University Hospital, Bergen, Norway
[5]Division of Child and Adolescent Psychiatry, Department of Psychiatry, Lausanne University Hospital, University of Lausanne, Lausanne, Switzerland
[6]Department of Child and Adolescent Psychiatry, King's College London, UK

CGT scores	Dropout analyses				
	T2 ADHD (n = 21)		T2 ADHD dropouts (n = 15)		t
	M	SD	M	SD	
T1 Risk adjustment	-0.17	0.85	-0.61	0.68	-0.89
T1 Delay aversion	0.33	0.97	-0.18	1.01	-0.46
T1 Reflection time	0.12	1.38	-0.20	0.78	-0.80
T1 Risk proneness	-0.12	0.96	0.16	0.73	0.95
FSIQ	93.43	7.00	91.47	5.84	-1.66
ADHD symptoms	31.21	8.47	30.70	8.18	-0.18

Note. ***p* < 0.01; **p* < 0.05. FSIQ = Full Scale IQ.

Supplemental Table 1. Between-group differences of the ADHD children participating in the follow-up assessment (T2) and the children with ADHD that dropped out after baseline (T1).

CGT scores	Between-group effects			Between-time point effects			Interaction effects^		
	F	*p*	h_p^2	*F*	*p*	h_p^2	*F*	*p*	h_p^2
Risk adjustment	8.91	0.004*	0.09	6.10	0.015	0.06	0.02	0.88	< 0.001
Delay aversion	4.46	0.037	0.047	18.16	< 0.001*	0.17	3.89	0.05	0.04
Reflection time	1.01	0.32	0.01	62.27	< 0.001*	0.41	0.73	0.39	0.01
Risk proneness	1.60	0.21	0.02	0.00	1.00	< 0.001	1.96	0.17	0.02

Note. *Bonferroni corrected *p* level < 0.013. ^= interaction effects between ADHD and time points. No covariates included. df = 1, 90.

Supplemental Table 2. The group effects on the decision parameters from the CGT over two time points.

CGT scores	Between-group effects			Between-time point effects			Interaction effects^		
	F	*p*	h_p^2	*F*	*p*	h_p^2	*F*	*p*	h_p^2
Risk adjustment	7.93	0.006*	0.08	0.00	0.96	< 0.001	0.02	0.89	< 0.001
Delay aversion	3.22	0.08	0.04	1.99	0.16	0.02	4.64	0.03	0.05
Reflection time	1.58	0.21	0.02	15.11	< 0.001*	0.15	0.74	0.39	0.01
Risk proneness	1.18	0.28	0.01	3.25	0.08	0.04	2.27	0.14	0.03

Note. *Bonferroni corrected *p* level < 0.013. ^ = interaction effects between ADHD and time points. Age and gender were included as covariates. df = 1, 88.

Supplemental Table 3. The group effects on the decision parameters from the CGT over two time points when adjusting for the effects of age and gender.

		Whole sample (N = 47)							
		2.	3.	4.	5.	6.	7.	8.	Age
1.	T1 Risk adjustment	0.36*	-0.43**	-0.26	-0.18	-0.17	-0.17	-0.15	0.10
2.	T2 Risk adjustment		-0.33*	-0.37*	-0.08	-0.47**	0.31*	-0.11	0.19
3.	T1 Delay aversion			0.34*	-0.13	0.21	-0.39**	0.22	-0.43**
4.	T2 Delay Aversion				-0.09	0.15	-0.09	-0.12	-0.36
5.	T1 Reflection time					0.52**	0.10	-0.12	0.14
6.	T2 Reflection time						-0.26	-0.08	-0.25
7.	T1 Risk proneness							-0.20	0.36*
8.	T2 Risk proneness								0.09
		ADHD (n = 21)							
		2.	3	4.	5.	6.	7.	8.	
1.	T1 Risk adjustment	0.51*	-0.35	0.043	-0.14	-0.09	0.34	-0.11	
2.	T2 Risk adjustment		-0.57**	-0.24	-0.13	-0.39	0.53*	-0.26	
3.	T1 Delay aversion			0.45*	-0.10	-0.13	-0.73**	0.18	
4.	T2 Delay Aversion				-0.13	0.11	-0.42	0.10	
5.	T1 Reflection time					0.53*	0.06	-0.14	
6.	T2 Reflection time						-0.20	-0.20	
7.	T1 Risk proneness							0.04	
8.	T2 Risk proneness								
		Controls (n = 26)							
		2.	3.	4.	5.	6.	7.	8.	
1.	T1 Risk adjustment	0.20	-0.36	-0.45*	-0.27	-0.10	-0.46*	-0.03	
2.	T2 Risk adjustment		-0.07	-0.46*	-0.05	-0.51**	0.24	0.08	
3.	T1 Delay aversion			0.29	-0.21	0.11	-0.18	0.08	
4.	T2 Delay Aversion				-0.06	0.22	0.12	-0.27	
5.	T1 Reflection time					0.56**	-0.05	-0.14	
6.	T2 Reflection time						-0.35	-0.19	
7.	T1 Risk proneness							0.32	
8.	T2 Risk proneness								

Note. ***p* < .01: **p* < .05.

Supplemental Table 4. Intercorrelations between the decision parameters from the CGT. The correlation between the CGT parameters and age are also included.

CGT scores	Gender					Group analysis
	Girls		Boys			
	M	SD	M	SD		*t*
T1 Risk adjustment	-0.10	0.85	-0.32	0.89		-0.82
T2 Risk adjustment	0.17	1.01	0.26	1.04		0.28
T1 Delay aversion	0.27	0.83	0.44	1.11		0.56
T2 Delay aversion	-0.39	0.60	-0.37	0.96		0.05
T1 Reflection time	0.80	0.85	0.48	0.93		-1.15
T2 Reflection time	-0.51	0.52	-0.69	0.50		-1.17
T1 Risk proneness	-0.30	1.05	0.19	1.08		1.51
T2 Risk proneness	-0.56	1.11	0.30	0.63		3.42**

Note. **$p < 0.01$; *$p < 0.05$.

Supplemental Table 5. The effect of gender on the decision parameters from the CGT.

CBCL scores:	Age
T1 Social problems	-0.07
T2 Social problems	-0.16
T1 Anxiety/depression problems	-0.10
T2 Anxiety/depression problems	-0.28
T1 Conduct problems	-<0.001
T2 Conduct problems	-0.08

Note. None of the correlations was significant at $p < 0.05$.

Supplemental Table 6. The correlation between the CBCL scores and age at T1 and T2.

Flow Chart for Sample Inclusion.

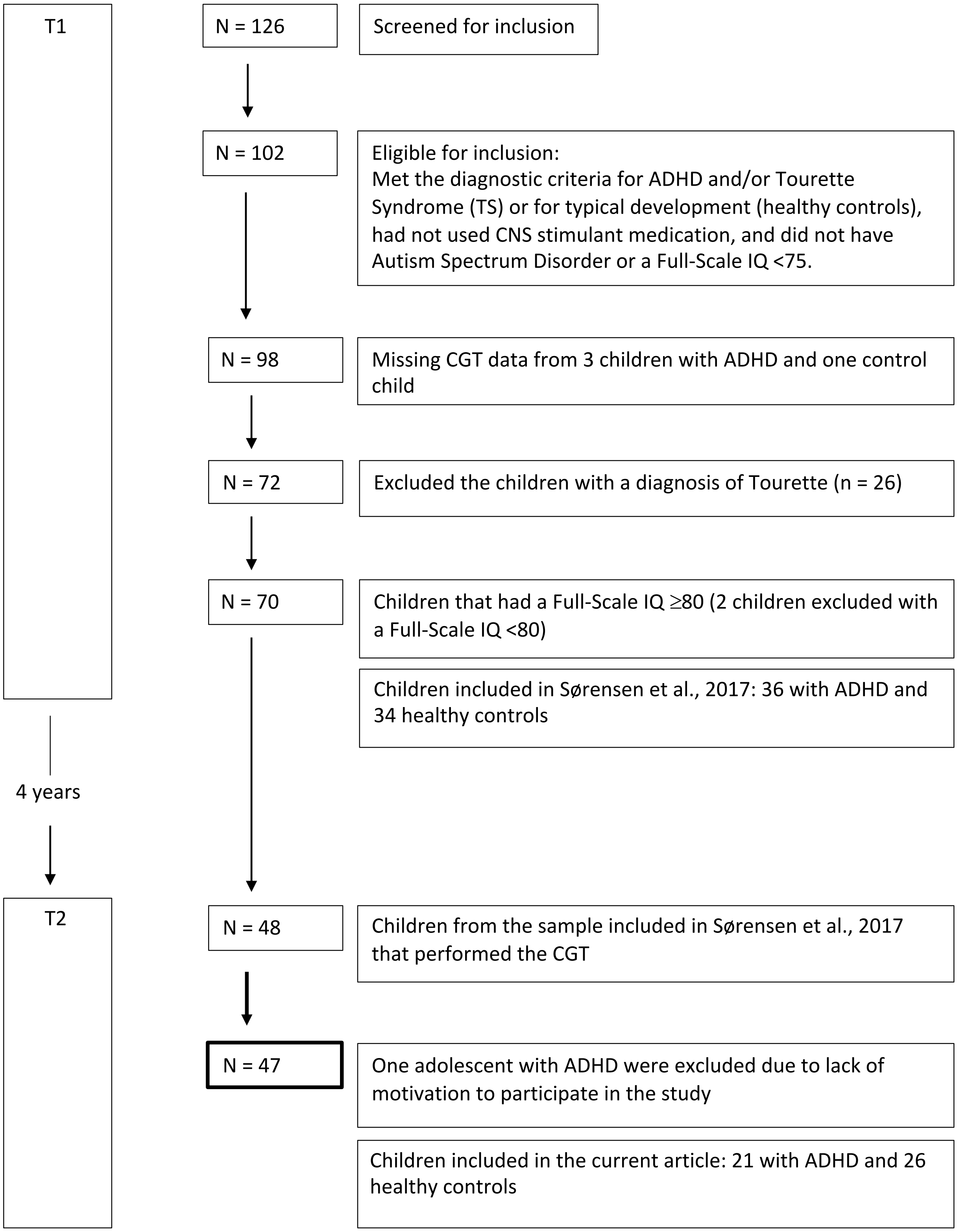

Supplemental Figure 1. A flowchart showing step-by-step information on the inclusion of participants in the current study ending up in the final sample included in the current study.
